# A comprehensive pre‐clinical treatment quality assurance program using unique spot patterns for proton pencil beam scanning FLASH radiotherapy

**DOI:** 10.1002/acm2.14400

**Published:** 2024-06-03

**Authors:** Pingfang Tsai, Yunjie Yang, Mengjou Wu, Chin‐Cheng Chen, Francis Yu, Charles B. Simone, Jehee Isabelle Choi, Wolfgang A. Tomé, Haibo Lin

**Affiliations:** ^1^ New York Proton Center New York New York USA; ^2^ Department of Radiation Oncology Memorial Sloan Kettering Cancer Center New York New York USA; ^3^ Department of Radiation Oncology Montefiore Medical Center and Albert Einstein College of Medicine Bronx New York USA

**Keywords:** proton, quality assurance, ultra‐high dose rate

## Abstract

**Background:**

Quality assurance (QA) for ultra‐high dose rate (UHDR) irradiation is a crucial aspect in the emerging field of FLASH radiotherapy (FLASH‐RT). This innovative treatment approach delivers radiation at UHDR, demanding careful adoption of QA protocols and procedures. A comprehensive understanding of beam properties and dosimetry consistency is vital to ensure the safe and effective delivery of FLASH‐RT.

**Purpose:**

To develop a comprehensive pre‐treatment QA program for cyclotron‐based proton pencil beam scanning (PBS) FLASH‐RT. Establish appropriate tolerances for QA items based on this study's outcomes and TG‐224 recommendations.

**Methods:**

A 250 MeV proton spot pattern was designed and implemented using UHDR with a 215nA nozzle beam current. The QA pattern that covers a central uniform field area, various spot spacings, spot delivery modes and scanning directions, and enabling the assessment of absolute, relative and temporal dosimetry QA parameters. A strip ionization chamber array (SICA) and an Advanced Markus chamber were utilized in conjunction with a 2 cm polyethylene slab and a range (*R*_80_) verification wedge. The data have been monitored for over 3 months.

**Results:**

The relative dosimetries were compliant with TG‐224. The variations of temporal dosimetry for scanning speed, spot dwell time, and spot transition time were within ± 1 mm/ms, ± 0.2 ms, and ± 0.2 ms, respectively. While the beam‐to‐beam absolute output on the same day reached up to 2.14%, the day‐to‐day variation was as high as 9.69%. High correlation between the absolute dose and dose rate fluctuations were identified. The dose rate of the central 5 × 5 cm^2^ field exhibited variations within 5% of the baseline value (155 Gy/s) during an experimental session.

**Conclusions:**

A comprehensive QA program for FLASH‐RT was developed and effectively assesses the performance of a UHDR delivery system. Establishing tolerances to unify standards and offering direction for future advancements in the evolving FLASH‐RT field.

## 1 INTRODUCTION

Recent preclinical studies^1^, ^2^, ^3^, ^4^, ^5^, ^6^, ^7^, ^8^, ^9^, ^10^ and human trials^11^, ^12^ have investigated FLASH radiotherapy (FLASH‐RT), a novel approach involving ultra‐high dose rate (UHDR) delivery (≥40 Gy/s). FLASH‐RT shows potential in reducing radiation‐induced normal tissue toxicities while maintaining comparable tumor control efficacy to conventional dose‐rate radiotherapy (0.01–0.4 Gy/s).^13^, ^14^, ^15^ The differential response between tumor and normal tissues under UHDR conditions, known as the FLASH effect,^16^, ^17^ has garnered significant interest.^18^, ^19^ However, the lack of readily available tools for UHDR measurement poses a challenge. Many existing quality assurance (QA) devices are inadequate for UHDR delivery, specifically for quantifying the dose rate for FLASH‐RT. Moreover, accurate dosimetry and dose rate quantification under UHDR conditions have become pressing issues for translating FLASH‐RT in clinical practice.^20^ A multi‐institutional dose verification survey conducted by Pedersen et al.^21^ in a preclinical setting revealed dose differences ranging from 12% to 42%. Standardization of experimental parameters is crucial to ensure valid cross‐experiment comparisons and reproducible results during radiobiology UHDR irradiations. Diffenderfer et al.^22^ emphasized the effort to determine optimized FLASH delivery parameters, including dose, dose rate, and spatiotemporal variation. Zou et al.^20^ explored current technology gaps and limitations in quality assurance, dosimetry, and UHDR delivery, underscoring specialized equipment and tailored protocols to facilitate the safe implementation of UHDR radiation treatments in clinical trials. Thus, the availability of a UHDR‐compatible dosimeter and standard QA parameters is critical for successful translation from preclinical studies to clinical applications.

Several studies^23^, ^24^, ^25^, ^26^, ^27^, ^28^ have established daily QA checks and jigs procedures following AAPM TG‐224 recommendations.^29^ However, a quantitative evaluation of the performance of the UHDR delivery system remains lacking. In particular, limited references are available for FLASH‐RT QA topics. In response to the absence of a consensus on UHDR delivery, a joined effort to standardize FLASH UHDR experiments (TG‐359) was placed by AAPM, Eastern Cooperative Oncology Group (ECOG), and the European College of Medical Physics (EFCOMP) in 2021. Furthermore, Spruijt et al.^30^ published a UHDR QA guideline based on a multi‐institutional consensus. The guidelines focus on temporal dosimetry, dose rate constancy, and suitable detectors, highlighting the unique requirements of UHDR proton. Several dosimetry studies^31^, ^32^, ^33^, ^34^, ^35^, ^36^ have compared potential UHDR dosimeters, and each dosimeter has limitations and trade‐offs regarding dose rate linearity and temporal resolution. In a recent study, Yang et al.^37^ investigated a two‐dimensional (2D) strip ionization chamber array (SICA) demonstrating desirable characteristics for proton pencil beam scanning (PBS) UHDR applications. The SICA boasts a high spatial and temporal resolution of 2 mm spacing between the center of the strips and a sampling rate of 50 µs, covering an active area of approximately 25.4 × 25.4 cm^2^. It has exhibited dose and dose rate linearity for UHDR irradiation up to 215 nA nozzle beam current, in addition to the ability to quantify the two‐dimensional dose rate, a crucial parameter for UHDR.

In this study, we have developed a comprehensive pre‐treatment QA program for PBS proton UHDR delivery to monitor the performance and achievability of the pre‐clinical delivery system. An all‐in‐one delivery pattern was designed, and the SICA detector was employed for measurement. The following sections will describe the pre‐treatment QA program and present its results. Appropriate tolerances suitable for UHDR pre‐treatment QA are proposed with long‐term monitoring data. This comprehensive QA program can serve as a future reference for the FLASH‐RT community.

## 2 METHODS

This study utilized a cyclotron‐based ProBeam proton therapy system (Varian Medical Systems Inc., Palo Alto, CA) with a fixed horizontal beamline configuration in a dedicated research room. In the UHDR mode, a scanned 250 MeV proton beam can be delivered at a nozzle beam current up to 215 nA. The pre‐treatment QA items for FLASH‐RT can be categorized into the following categories: (i) safety, (ii) laser, and (iii) beam quality checks, specifically focusing on absolute, relative, and temporal dosimetry. The appropriate tolerances for pre‐treatment QA in FLASH‐RT were established.

### 2.1 Safety check

Prior to UHDR irradiation, several safety checks were performed to ensure the proper functionality of door interlocks, audio and visual monitoring systems, and radiation indicators. Radiation safety was confirmed by pressing the in‐room search button to trigger the appropriate reaction and validating the corresponding interlock activation on the radiation indicators.

### 2.2 Laser check

The alignment of lasers was verified using the wall markings around the treatment area, which were established during the acceptance test. Additionally, the coincidence of the lasers with the isocenter was confirmed. As the research room lacks an image‐guided system for additional positioning guidance, the setup of the measurement device for beam quality QA relied on the lasers. One of the sampling data from the center location of SICA was extracted, and the peak spot position of this sample was analyzed to ensure there were no significant positional deviations during the observation period.

### 2.3 Beam quality check

**FIGURE 1:**
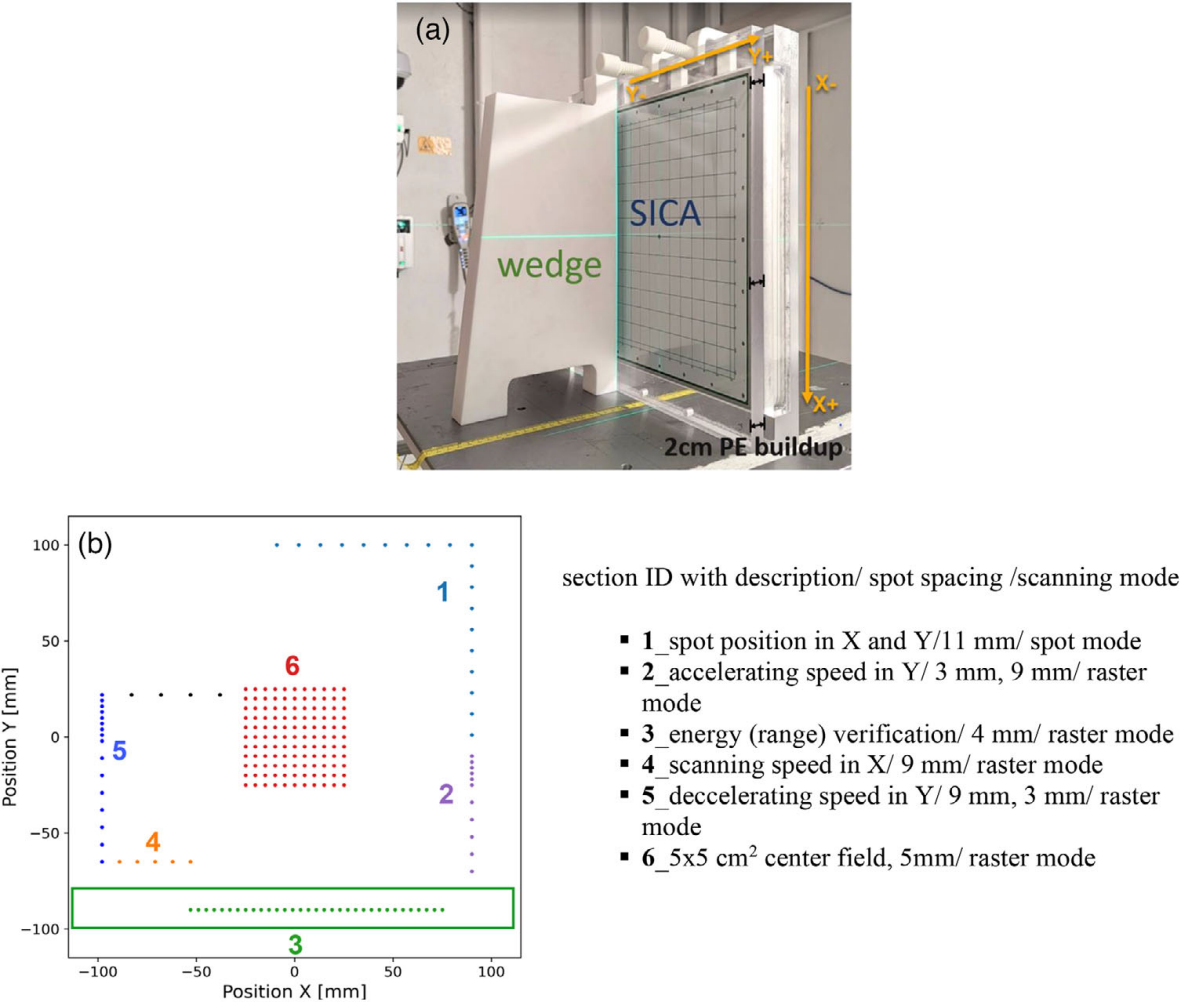
The FLASH‐RT pre‐treatment QA setup and spot map. (a) The measurement setup with 2 cm polyethylene (PE) slab, SICA detector, and a range verification wedge. (b) The beam's eye view spot pattern for beam quality checks includes a detailed description of the settings for each section. (b) is displayed 90 degrees counterclockwise from what is shown in (a). The green box area indicated the place for the range verification wedge.

Previous studies have demonstrated the excellent linearity in dose and dose rate measured by the Advanced Markus chamber (PTW, Freiburg, Germany) and the SICA detector (Liverage Biomedical Inc., Hsinchu, Taiwan).^37^, ^38^, ^39^, ^40^ Hence, these two dosimeters were chosen for this study. The sampling frequency (20 kHz) of SICA is preset by the manufacturer in the hardware to optimally balance signal processing capabilities with the anticipated characteristics of UHDR experiments. As illustrated in Figure 1a, the isocenter was aligned with the surface of a 2 cm polyethylene (PE) slab placed in front of the SICA detector. An Advanced Markus chamber was positioned directly downstream from the center of the SICA detector to ensure consistent detector response throughout the observation period. An all‐in‐one proton pre‐treatment QA pattern was designed to assess the consistency of absolute, relative, and temporal dosimetry in UHDR delivery. The pattern was designed to deliver the lowest monitor unit (MU) per spot for the given beam current, minimizing the radiation dose for this test. The delivery sequence of the QA pattern is structured a spiral fashion, progressing from Sections 1–6, as illustrated in Figure 1b.

A 250 MeV single‐energy‐layer spot map was delivered using the proton PBS technique under UHDR conditions. The pattern consisted of a central 5 × 5 cm^2^ field with 50 MUs per spot, along with several spot‐scanning lines in the peripheral region with 60 MUs per spot. The peripheral lines were asymmetrically arranged around the center, with varying spot spacings covering an approximate 20 × 20 cm^2^ field. The most inferior peripheral line was positioned to assess the proton range of the 250 MeV proton beam employing a polytetrafluoroethylene (PTFE) wedge. The wedge placement relative to the SICA detector is illustrated in Figure 1b from a beam's eye view in IEC scale.

This study examined the spot pattern in various sections using both spot and raster delivery modes. In the spot mode, the proton beam is turned off while transitioning to the next spot, applicable for spots with spacing greater than the spot spacing threshold. On the other hand, in the raster mode, the proton beam remains active during transitions to the next spot position, applicable for spots with spacing equal or less than the spot spacing threshold. In this study, the spot spacing threshold was set at 10 mm for both the X and Y directions, allowing the delivery system to switch between raster and spot mode during UHDR deliveries depending on the different sections of the patterns.

The detailed design for the testing functions and spot spacing (SS) in each section is presented in Figure 1b. The functions of each section are described as follows:

1. **Section** **1**: This section assesses spot position and spot size accuracy in the IEC‐X and IEC‐Y scanning directions.
2. **Section** **2**: This section aims to evaluate the accelerating speed, which involves a combination of SS in 3 and 9 mm, as well as spot dwell time and transition time in the Y direction. (Note: The spot scanning speed is higher in the IEC‐Y direction than in the IEC‐X direction.)
3. **Section** **3**: In this section, the energy (range) verification of the proton beam is performed.
4. **Section** **4**: The scanning speed, spot dwell time, and transition time in the X direction are examined in this section.
5. **Section** **5**: This section aims to assess the decelerating speed, which includes a combination of SS in 9 and 3 mm, spot dwell time, and transition time in the Y direction.
6. **Section 6**: A uniform field of 5 × 5 cm^2^ with 5 mm SS is scanned in the Y direction to monitor the absolute dose, dose rate, and field properties.

The same pattern was delivered three times per beam current each quality assurance day, with the requested nozzle beam currents of 5 and 215 nA. A total of 48 irradiations at each nozzle beam current were performed over a 3‐month period. Measurements using a 5 nA beam current aimed to characterize the point dose response of the Advanced Markus chamber under non‐UHDR conditions. The 215 nA setting was selected to evaluate the delivery system's performance at the highest attainable UHDR, ensuring its reliability under the most demanding conditions. The SICA detector was utilized to capture the dynamics of UHDR, providing two‐dimensional information. The baselines of the quality assurance items were determined by averaging the measurements from the first three days. The only exception was the range baseline, which was obtained through measurements using a multi‐layer ionization chamber (Zebra, IBA dosimetry GmbH, Schwarzenbruck, Germany).

### 2.4 Data analysis

For absolute dosimetry, the beam‐to‐beam output variation was calculated by subtracting the minimum output of a given day from that day's maximum output, then dividing it by the daily averaged output. The day‐to‐day output variation was determined by subtracting the minimum output from the maximum output during the observation period and dividing it by the baseline. The absolute output was measured using an Advanced Markus chamber under 5 and 215 nA beam currents following the IAEA TRS‐398 protocol.^41^ The method employed for reconstructing the SICA detector's two‐dimensional (2D) dose distribution was based on the description provided by Yang et al.^30^ The absolute dose for the SICA detector was obtained in the uniform area of the central 5 × 5 cm^2^ field. The ratio of output measured by the SICA detector and the Advanced Markus chamber was investigated.

For relative dosimetry, properties of the central 5 × 5 cm^2^ field, such as flatness, symmetry and penumbra were calculated. Spot position accuracy was determined by comparing the spot peak location with the planned locations. The spot size was defined as the width parameter (σ) of a Gaussian distribution fit to the measured spot profile. In terms of energy range analysis, the proton range (*R*_80_) was defined as the depth of the 80% distal dose fall‐off in water equivalent thickness.

**FIGURE 2:**
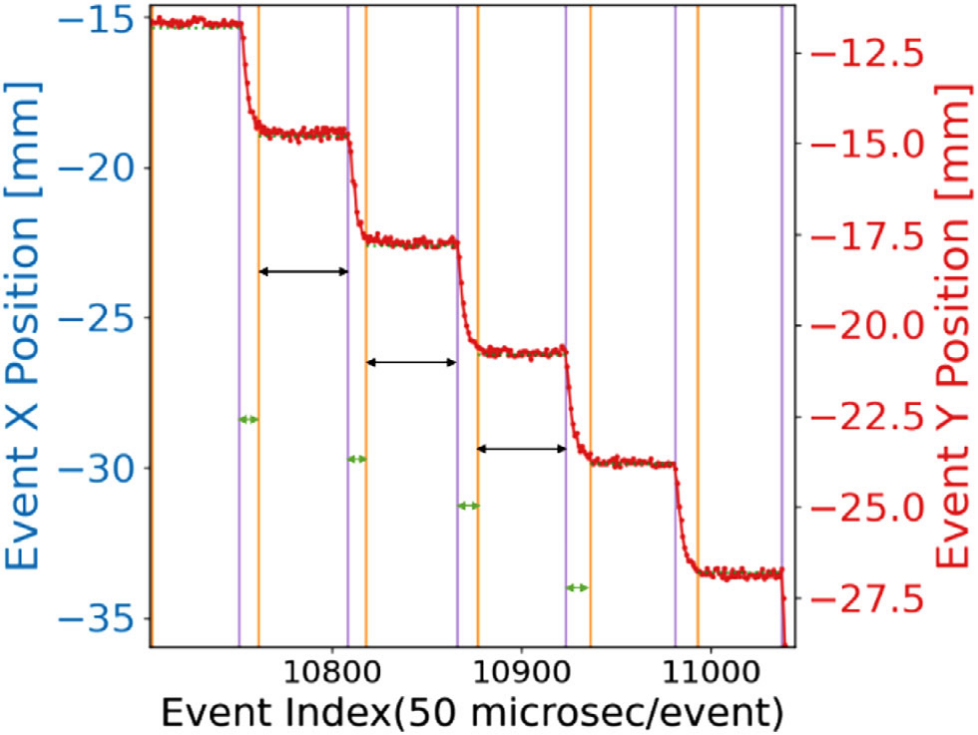
The spot X and Y position as a function of the event index measured by the SICA. The long‐black arrow time windows (constant x and y) show the spot dwell time. The narrow green arrows (x and y transition) show the spot transition times.

For temporal dosimetry, the spot dwell and transition time intervals were visually inspected, with Figure 2 illustrating the X and Y positions in relation to the event index, serving as a proxy of the elapsed time. In Figure 2, the start of a spot dwell time was indicated by an orange vertical line, while a purple vertical line marked the end of a spot dwell time. The spot transition time was defined as the interval between the end time of the previous spot (j−1) dwell and the start time of the current spot (j) dwell. With the known travel distance from the planned position and the spot transition time, the scanning speed (mm/ms) was calculated by dividing the travel distance by the spot transition time. For the dose rate analysis, the local average dose rate (LADR) defined by Folkerts et al.^42^ was adopted. For the LADR, $D(\vec{x})$ is the dose at the voxel of interest (VOI), and the dose threshold, $d^{†}$, was selected to be $d^{†}=\;0.05D\left(\vec{x}\right)$. After obtaining the LADR for each voxel, a histogram was generated to visualize the distribution of LADR values across all voxels. Within this distribution, the mode, defined as the most frequently occurring LADR value, was identified as the ridge dose rate.^39^ This choice is particularly advantageous over the mean or median LADR in this context due to the complex nature of the dose rate map. The ridge dose rate, being the most recurrent value, is less susceptible to these variations and offers a more consistent and reliable indicator of dose rate consistency analysis.

## 3 RESULTS

**FIGURE 3:**
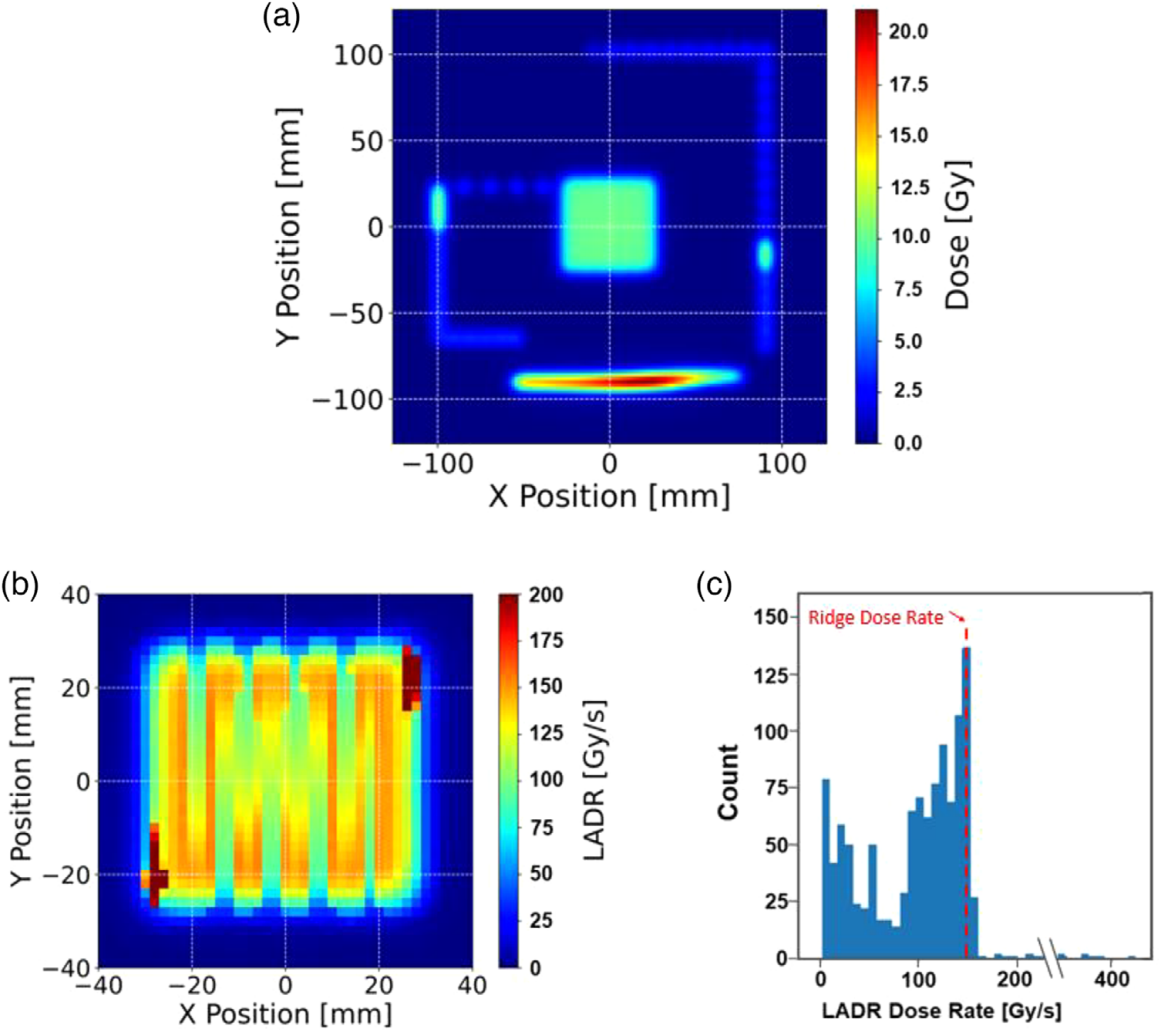
The SICA measurement for QA testing, including (a) the reconstructed 2D absolute dose map of SICA measurement. (b) depict the corresponding 2D LADR map of the central 5 × 5 cm^2^ and (c) the voxel value distribution histogram.

The reconstructed 2D absolute dose distribution of SICA measurement is displayed in Figure 3a. Figure 3b,c shows the associated 2D LADR map and the voxel value distribution histogram of the central 5 × 5 cm^2^ region, respectively.

### 3.1 Absolute dosimetry

**FIGURE 4:**
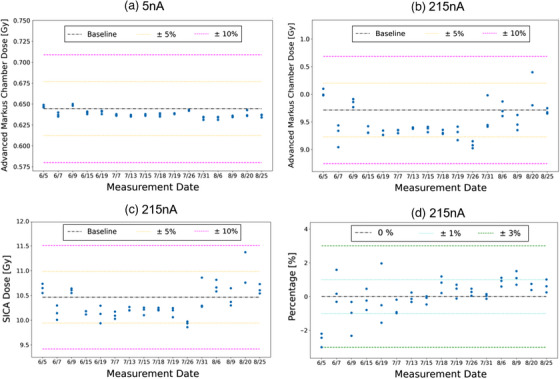
Absolute dose trend analysis with measurement date on the horizontal axis. (a) Absolute dose (Advanced Markus Chamber, 5 nA nozzle beam current). (b) Absolute dose (Advanced Markus Chamber, 215 nA nozzle beam current). (c) Absolute dose (SICA, 215 nA nozzle beam current). (d) The ratio of absolute dose measured by SICA and Advanced Markus Chamber.

The trending analysis was conducted for the Advanced Markus chamber and SICA detector in the central 5 × 5 cm^2^ region, as shown in Figure 4. Figure 4a presents the beam‐to‐beam variation on the same day for a 5 nA beam current, which was observed to be 0.47%, and the day‐to‐day variation at the same beam current was found to be 2.39%. In contrast, for 215 nA nozzle beam current, the beam‐to‐beam variation on the same day was 2.14%, whereas the day‐to‐day variation was 9.69%, as illustrated in Figure 4b. Notably, both the SICA detector and the Advanced Markus chamber exhibited similar variations under 215 nA nozzle beam current, as showed in Figure 4b,c. The observed variations in this study were also reported by another research group.^43^ The beam‐to‐beam variations observed on the same day or within the same experimental session were smaller compared to the day‐to‐day fluctuations. Figure 4d shows that when the percentage ratio of the SICA and Advanced Markus chamber was calculated, the extent of variation was 0.5% on the same day and up to 3% for day‐to‐day variation. This finding suggests that both detector responses remained consistent during the observation period in this study.

### 3.2 Relative dosimetry

#### 3.2.1 Field properties

The lateral penumbra of the central 5 × 5 cm^2^ field was determined to be 6.24 ± 0.16 mm and 6.16 ± 0.16 mm in the X‐ and Y‐directions, respectively. Overall, the variation of lateral penumbra was less than 0.1 mm. The symmetry variation was found to be 0.21 ± 0.13% and 0.11 ± 0.06% in the X‐ and Y‐directions, respectively, whereas the flatness variation was 4.88 ± 0.24% and 4.65 ± 0.22% in the X‐ and Y‐directions, respectively. All field properties complied with the suggested tolerances in TG‐224.

#### 3.2.2 Spot position

**FIGURE 5:**
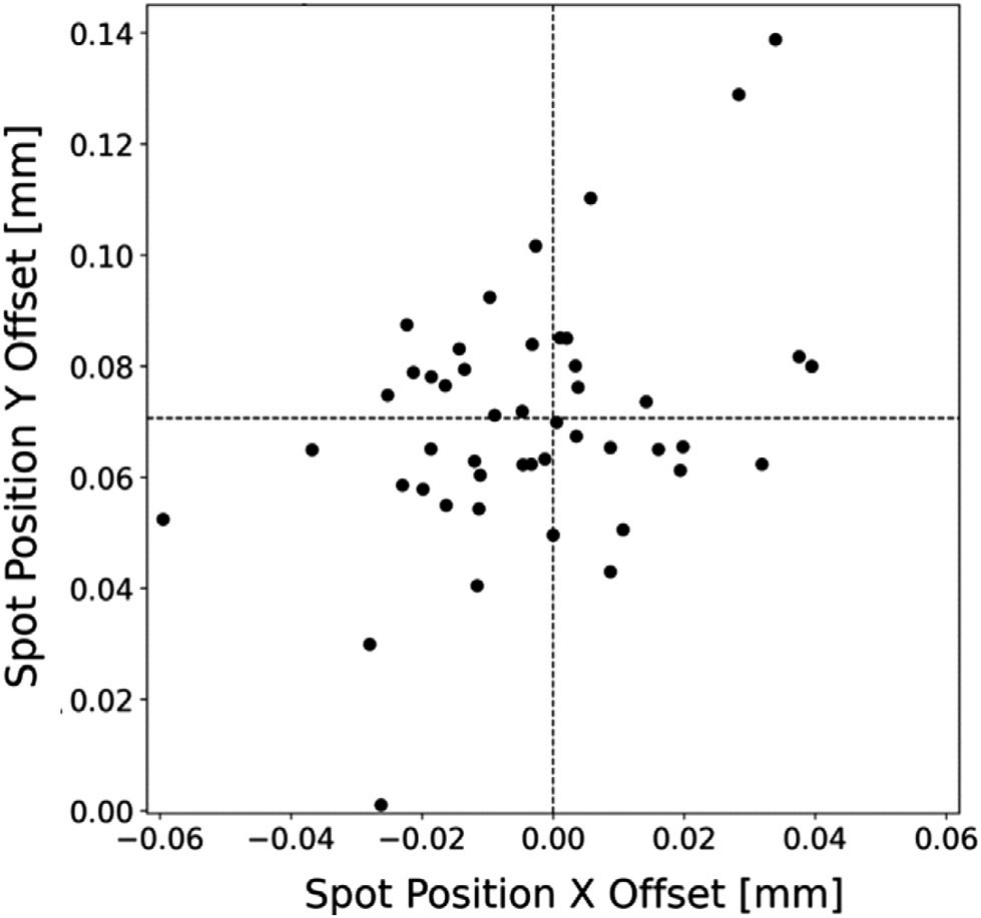
The relative spot position offsets with respect to the planned spot positions of 48 irradiations carried out over 3 months under 215 nA nozzle beam current.

Figure 5 illustrates the spot position accuracy with X‐ and Y‐offsets compared to the planned position over 3 months. The offsets in the X‐direction were within −0.002 ± 0.02 mm (ranging from −0.06 to 0.04 mm), whereas in the Y‐direction, the position offsets were 0.07 ± 0.02 mm (ranging from 0.001 to 0.14 mm). It is important to note that the spot positions offsets shown in Figure 5 represent the relative spot position to the center spot and do not include the effects of day‐to‐day variation in the setup with respect to the in‐room laser system or the discrepancy between the in‐room laser isocenter and the radiation isocenter. These offsets primarily reflect the machine's scanning performance, specifically the scanning magnet's control of the relative spot positioning under UHDR conditions.

#### 3.2.3 Spot size

**FIGURE 6:**
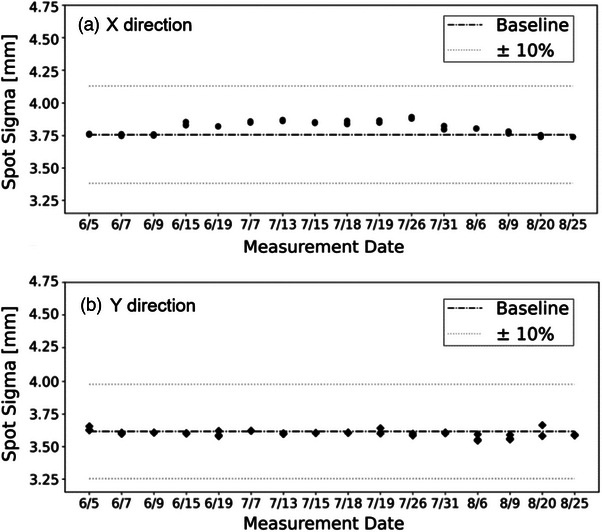
Trend analysis of spot size.

Figure 6 presents the trending analysis of spot size (σ), indicating variations of 3.8 ± 0.048 mm for σ_x_ and 3.6 ± 0.022 mm for σ_y_. The observed deviation from the baseline is deemed minimal, falling within the normal fluctuation range and well below the ± 10% tolerance level. Throughout the 3‐month period, the 95% confidence intervals for spot size deviations in both the X and Y directions were found to be less than 0.2 mm. This consistency underlines the delivery system's capability to maintain spot size accuracy effectively, even under UHDR conditions.

#### 3.2.4 Range verification

The range verification conducted using the wedge phantom showed excellent consistency. On the same day, the average deviations in R_80_ measured less than 0.3 mm, while the day‐to‐day variation reached up to 1.3 mm.

### 3.3 Temporal dosimetry

#### 3.3.1 Field dose rate

**FIGURE 7:**
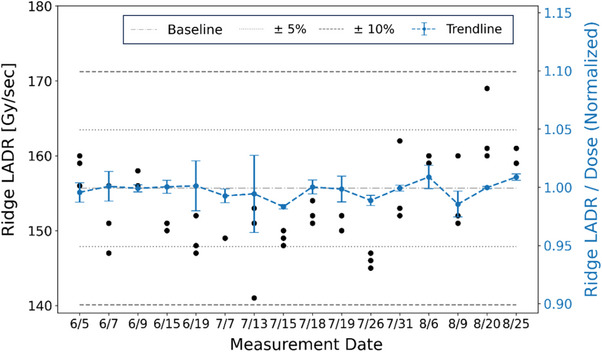
Trend analysis of ridge dose rate (left Y‐axis, black circle) and the normalized ridge LADR (right Y‐Axis, blue dash curve) with respect to absolute dose, presented with error bars indicating one standard deviation.

As shown in Figure 7, the ridge dose rate of the center 5 × 5 cm^2^ field exhibited a baseline value of 155 Gy/s, which achieve the UHDR level. A day‐to‐day variation of ± 5%, and fluctuations of up to 10% were observed over the observation period. However, after normalizing each dose rate and dose measurement to their respective baselines revealed a strong correlation between dose rate variations and absolute dose output (with a ratio close to 1). This implied that the variation largely depends on the time required to accomplish a particular delivery under UHDR conditions. As the dose is normalized (fixed), the blue trend line in Figure 7 shows how some days the fluctuations hover close to less than 1%, while on others, they can reach up to 5%.

#### 3.3.2 Scanning speed

**FIGURE 8:**
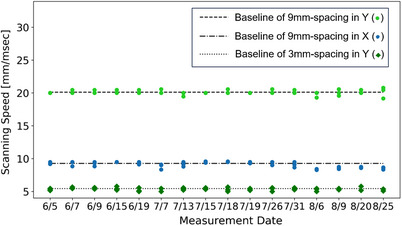
Scanning speed analysis.

Figure 8 presents the analysis of scanning speed, revealing the scanning speeds for 9 mm SS(Y), 9 mm SS(X), and 3 mm SS(Y) were within ± 1 mm/ms of the baseline values (20, 9, 5.5 mm/ms, respectively). Notably, for the Varian ProBeam system, the scanning speed in the Y direction (20 mm/ms) was faster compared to the X direction (9 mm/ms) for the same 9 mm SS. Notably, increasing the spot spacing from 3 to 9 mm nearly doubled the scanning speed in Y direction. This suggests that using the raster mode in the Y direction is preferable for achieving higher UHDR delivery through faster scanning. Furthermore, an increase in spot spacing within the same scanning direction corresponded to a higher scanning speed before the maximum scanning speed is reached. These findings suggest that for a predetermined field size and spot spacing, the chosen scanning speed and direction can significantly impact the delivery efficiency.

#### 3.3.3 Spot dwell and transition time

**FIGURE 9:**
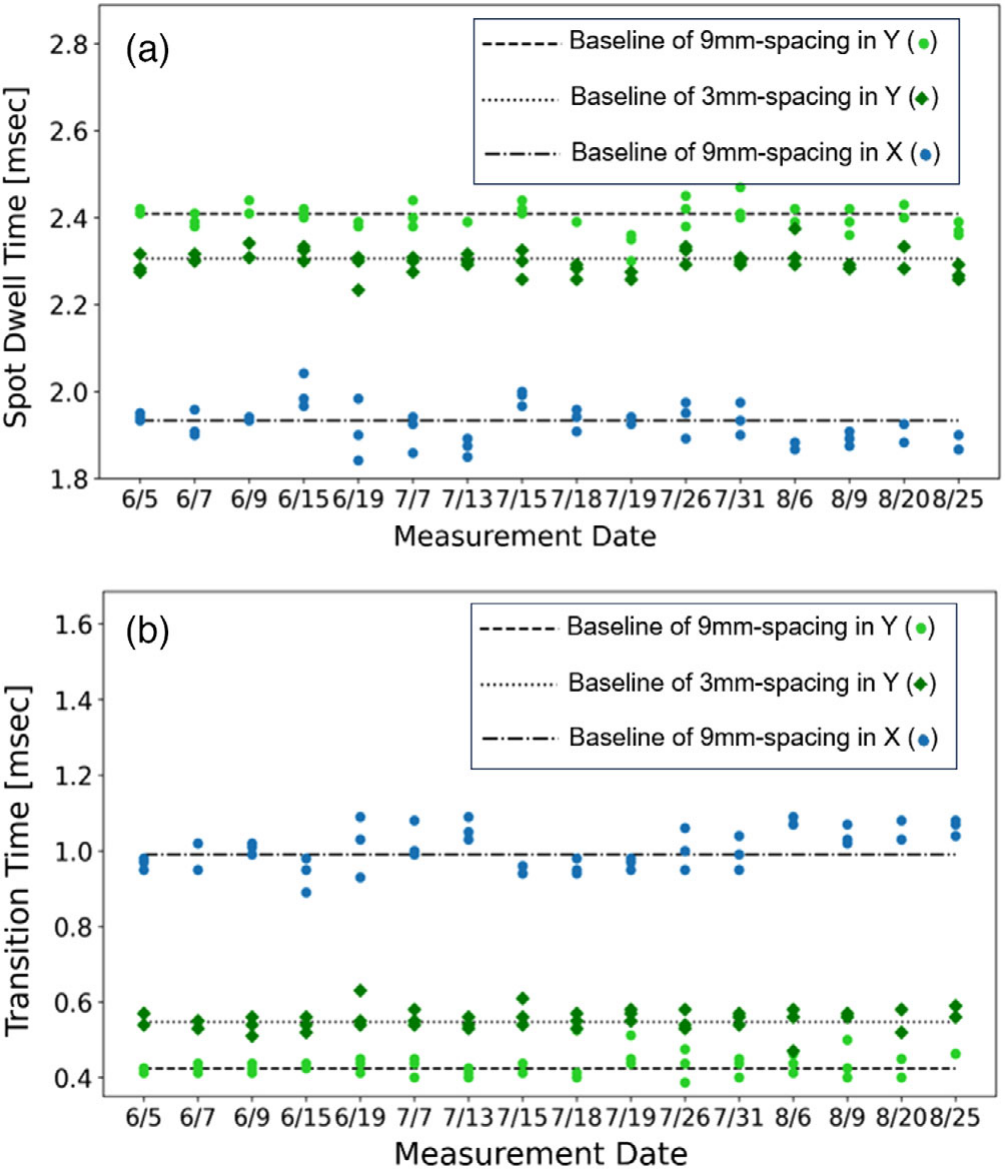
The spot dwell time and transition time trend analysis: (a) spot dwell time (b) transition time.

Figure 9a,b shows the trend analysis for spot dwell time and transition time, respectively, with both demonstrating variations within ± 0.2 ms. For the 9 mm SS, the X direction exhibited more variability in both dwell and transition times compared to the Y direction. When analyzing the same scanning direction, a comparison between the 9 and 3 mm SS data points showed that a smaller SS was associated with longer spot dwell times and shorter transition times, assuming identical monitor units (MUs) per spot. Additionally, when comparing the X and Y scanning directions, the X direction consistently showed shorter dwell times and longer transition times than the Y direction. Within the parameters of SS and scanning direction used in this study, dwell times were observed to range from 1.9 to 2.4 ms, while transition times varied from 0.4 to 1 ms.

### 3.4 Summary

A comprehensive pre‐treatment QA program for FLASH‐RT was established, encompassing safety, laser, and beam quality checks. Based on the achievability observed during the long‐term observation period of this study, suggested tolerances were summarized in Table 1.

**TABLE 1:** Comprehensive pre‐treatment QA tolerances for proton PBS FLASH radiotherapy.

Quality assurance items	Tolerances
**Safety checks**	
Door warning	Functional
Intercom	Functional
Search button	Functional
Beam pause	Functional
Beam on indicator	Functional
**Laser checks**	
Laser Sup‐Inf	±2 mm
Laser Lt‐Rt	±2 mm
Laser Ant‐Post	±2 mm
**Beam quality checks**	
**Absolute dosimetry**	
Output constancy (same day)	±2% / ± 5%^a^
Output constancy (day‐to‐day)	±3% / ± 10%^a^
**Relative dosimetry**	
Field symmetry	±1%^b^
Field flatness	±2%^b^
Field lateral penumbra	±2 mm^b^
Spot position	±1 mm^b^
Spot size	±10%^b^
Range verification	±1 mm^b^
**Temporal dosimetry**	
Field dose rate (same day)	±2%/±5%^a^
Field dose rate (day‐to‐day)	±3%/±10%^a^
Scanning speed	±1 mm/ms
Spot dwell time	±0.2 ms
Spot transition time	±0.2 ms

^a^ Proposed tolerance for (non‐saturated monitor chamber/saturated monitor chamber) under ultra‐high dose rate delivery.

^b^ Compliance with TG‐224.

## 4 DISCUSSION

A comprehensive pre‐treatment QA program was successfully developed and implemented for the preclinical proton UHDR RT research beamline. This study provided long‐term monitoring of dose, dose rate, and spatiotemporal accuracy of spot deliveries for UHDR. The spot position QA results confirmed the precision of the nozzle scanning magnets in delivering radiation to the designated spot positions under UHDR conditions. Raster mode was specifically selected for temporal dosimetry because it can achieve higher dose rates in UHDR RT. Moreover, the Y‐direction scanning was chosen for the QA tests, as it features a faster scanning speed than the X‐direction in the ProBeam proton system. This deliberate choice facilitated a comprehensive evaluation of the capability and control under more rigorous and demanding conditions for the UHDR delivery system. The various spot spacing, ranging from 3 to 9 mm, covered practical clinical scenarios. The observed correlation between dose and dose rate in the trend analysis in Figure 7 emphasizes the importance of considering both factors in QA programs.

The machine monitor chamber exhibited saturation behavior at ultra‐high dose rates, causing significant output and beam current (dose rate) variations compared to deliveries under conventional dose rates.^30^, ^38^, ^39^, ^43^ The strong dependence on the cyclotron output current led to dose and dose rate variations. The suggested tolerances for UHDR output constancy flag errors or failures in UHDR delivery and should be adjusted based on the dosimeters and delivery system. Daily calibration through the pre‐treatment QA program, followed by scaling of monitor units to account for day‐to‐day variations, is strongly recommended and mandatory for accurate treatment. An in vivo dosimetry system could capture beam‐to‐beam output fluctuations and retrospectively estimate the true delivered dose.

The dose rate is critical in the FLASH‐RT QA program, as it depends on the delivered dose and time. Monitoring the temporal characteristics of the PBS delivery, including spot dwell and transition times, is essential to ensure high‐quality treatment. This study presented high consistent (variations < 0.2 ms) spot dwell time and transition time over a long‐term observation period. The impact of scanning speed variations on dose rate was also found to be relatively minimal by Huang et al.,^44^ in which a 10% discrepancy in scanning speed results in a 1% effect on the resultant dose rate for typical clinical plans.

Joined efforts from vendors and users are crucial in developing specific dosimeters for UHDR RT. Due to the limited availability of the UHDR delivery system and fast‐evolving QA dosimeter, it becomes evident that comprehensive dosimeter tests across multiple institutions and delivery systems are essential. Moreover, the availability of a FLASH‐compatible monitor chamber in future clinical conditions is expected to reduce dose and dose rate fluctuations. Standardizing critical temporal parameters for FLASH‐RT across different cyclotron‐based or pencil beam scanning delivery systems should be a priority to advance the field.

## 5 CONCLUSIONS

A comprehensive pre‐treatment QA program for FLASH‐RT was developed, incorporating both the existing conventional QA items and the essential temporal QA items for UHDR. Long‐term monitoring and evaluation of machine performance have demonstrated that the integrated QA pattern effectively assesses the performance of a UHDR delivery system using clinically relevant parameters. By proposing tolerances and establishing a unified standard, this program not only sets a benchmark for FLASH‐RT but also offers direction for ongoing and future advancements in this evolving field.

## AUTHOR CONTRIBUTION

The authors contributed to the paper as follows: study design: Pingfang Tsai, Yunjie Yang, Chin‐Cheng Chen, and Haibo Lin; data analysis: Mengjou Wu; measurement data collection: Pingfang Tsai, Chin‐Cheng Chen; phantom design: Francis Yu; manuscript preparation and revision: Pingfang Tsai, Charles B. Simone, Isabelle Choi, Wolfgang A. Tomé, and Haibo Lin. All authors reviewed the results and approved the final version of the manuscript.

## CONFLICT OF INTEREST STATEMENT

The authors declare No conflicts of interest.
